# Biofilm and metallo beta-lactamase production among the strains of *Pseudomonas aeruginosa* and *Acinetobacter* spp. at a Tertiary Care Hospital in Kathmandu, Nepal

**DOI:** 10.1186/s12941-017-0245-6

**Published:** 2017-11-02

**Authors:** Bandana Baniya, Narayan Dutt Pant, Sanjeev Neupane, Saroj Khatiwada, Uday Narayan Yadav, Nisha Bhandari, Rama Khadka, Sabita Bhatta, Raina Chaudhary

**Affiliations:** 1 Department of Microbiology, Kantipur College of Medical Science, Sitapaila, Kathmandu, Nepal; 2 Department of Microbiology, Grande International Hospital, Dhapasi, Kathmandu, Nepal; 3 Central Department of Microbiology, Tribhuvan University, Kirtipur, Kathmandu, Nepal; 4 Department of Biochemistry, Modern Technical College, Lalitpur, Kathmandu, Nepal; 5 Forum for Health Research and Development, Dharan, Nepal; 6 Department of Biotechnology, University of Madras, Chennai, India; 7 Department of Microbiology, Nepalese Army Institute of Health Sciences, Syanobharyang, Kathmandu, Nepal

**Keywords:** Biofilm, MBL, *Pseudomonas aeruginosa*, *Acinetobacter* spp.

## Abstract

**Introduction:**

*Pseudomonas aeruginosa* and *Acinetobacter* spp. are found to be associated with biofilm and metallo-β-lactamase production and are the common causes of serious infections mainly in hospitalized patients. So, the main aims of this study were to determine the rates of biofilm production and metallo beta-lactamase production (MBL) among the strains of *Pseudomonas aeruginosa* and *Acinetobacter* spp. isolated from hospitalized patients.

**Methods:**

A total of 85 *P. aeruginosa* isolates and 50 *Acinetobacter* spp. isolates isolated from different clinical specimens from patients admitted to Shree Birendra Hospital, Kathmandu, Nepal from July 2013 to May 2014 were included in this study. The bacterial isolates were identified with the help of biochemical tests. Modified Kirby-Bauer disc diffusion technique was used for antimicrobial susceptibility testing. Combined disc diffusion technique was used for the detection of MBL production, while Congo red agar method and tube adherence method were used for detection of biofilm production.

**Results:**

Around 16.4% of *P. aeruginosa* isolates and 22% of the strains of *Acinetobacter* spp. were metallo β-lactamase producers. Out of 85 *P. aeruginosa* isolates, 23 (27.05%) were biofilm producers according to tube adherence test while, only 13 (15.29%) were biofilm producers as per Congo red agar method. Similarly, out of 50 *Acinetobacter* spp. 7 (14%) isolates were biofilm producers on the basis of tube adherence test, while only 5 (10%) were positive for biofilm production by Congo red agar method. Highest rates of susceptibility of *P. aeruginosa* as well as *Acinetobacter* spp. were seen toward colistin.

**Conclusion:**

In our study, biofilm production and metallo beta-lactamase production were observed among *Pseudomonas aeruginosa* and *Acinetobacter* spp. However, no statistically significant association could be established between biofilm production and metallo beta-lactamase production.

## Introduction

With the emergence of carbapenemase (mainly metallo-β-lactamase) producing bacterial strains, the clinical utility of carbapenem as reserve drug is under threat [1]. Such bacterial strains have emerged all over the world and show high-level resistance to all β-lactams except aztreonam [2]. Antimicrobial resistance associated with biofilm production presents a serious threat in clinical practice in case of biofilm associated infections [3, 4]. Biofilm producers produce an extracellular matrix of polysaccharides which act as a protective jacket for the bacteria within biofilms, preventing diffusion of antibiotics, immune cells and host proteins [5]. Thus, it is of utmost importance to screen for biofilm production among clinical isolates. The first step in the treatment of biofilm associated infections is the detection of biofilms and it needs to be incorporated as a routine laboratory procedure. Antibiotics used for the treatment of such infections should be directed against the biofilms rather than the planktonic forms [6]. *Pseudomonas aeruginosa* and *Acinetobacter* spp. are the common causes of life threatening infections mainly in hospitalized patients [7]. In addition, biofilm and metallo-β-lactamase production among these bacteria may present as serious problem to the treatment of the infections caused by them. In this study, we determined the rates of biofilm production and MBL production among the strains of *Pseudomonas aeruginosa* and *Acinetobacter* spp. Further, we determined the antimicrobial susceptibility patterns of these organisms.

## Methods

A total of 85 *P. aeruginosa* isolates and 50 *Acinetobacter* spp. isolates, isolated from different clinical specimens from patients admitted to Shree Birendra Hospital, Kathmandu, Nepal from July 2013 to May 2014 were included in this study. The bacterial isolates were identified on the basis of the microbiological procedures as described in the Bergey’s manual of determinative bacteriology and were further evaluated for MBL production by combined disk diffusion method using imipenem and imipenem/ethylenediaminetetraacetate discs. Similarly, biofilm production was detected by tube adherence method and Congo red agar method [8, 9]. Modified Kirby-Bauer disc diffusion technique was used for antimicrobial susceptibility testing following clinical and laboratory standards institute guidelines 2013. The bacteria showing resistance to at least three different classes of antibiotics were taken as MDR. Statistical package for the social sciences version 16.0. was used for data analysis. Chi square test was applied and *p* < 0.05 was considered significant.

## Results

Of the total 85 *P. aeruginosa* isolates, 14 (16.4%) were metallo β-lactamase producers, while of the 50 *Acinetobacter* spp. 11 (22%) were metallo β-lactamase producers.

Out of 85 *P. aeruginosa* isolates, 23 (27.05%) were biofilm producers according to tube adherence test, while only 13 (15.29%) were biofilm producers as per Congo red agar method. Similarly, out of 50 *Acinetobacter* spp. 7 (14%) isolates were biofilm producers on the basis of tube adherence test while, only 5 (10%) were positive for biofilm production by Congo red agar method. Of the total 14 MBL producing *P. aeruginosa* isolates, only 2 were biofilm producers on the basis of Congo red agar method, whereas 4 were biofilm producers as per tube adherence method. Similarly, of total 11 MBL producing strains of *Acinetobacter* spp., only 2 were biofilm producers on the basis of Congo red agar method, whereas 1 was biofilm producer as per tube adherence method. Highest rate of susceptibility of *P. aeruginosa* was seen toward colistin (83.5%) followed by netilmycin (71.9%). Lowest rate of susceptibility was seen toward cefepime (22.4%). Highest rate of susceptibility of *Acinetobacter* spp. was seen toward colistin (74%) followed by netilmicin (70%). Lowest rate of susceptibility was seen toward ceftriaxone (6%) (Table 1). Of the total *P. aeruginosa* isolates, 56 (65.8%) were multidrug resistant, while of the total *Acinetobacter* spp. isolates, 45 (90%) were multidrug resistant. Higher rates of drug resistance were seen among biofilm producers in comparison to biofilm non producers but the correlation was statistically insignificant.

**Table 1:** Antibiotic susceptibility patterns of *Acinetobacter* spp. and *P. aeruginosa*

Antibiotics	Susceptible	
	*Acinetobacter* spp. (%)	*P. aeruginosa* (%)
Piperacillin–tazobactam	24	45.9
Ceftazidime	14	49.4
Cefepime	16	22.4
Imipenem	56	69.4
Gentamicin	22	48.2
Amikacin	22	50.6
Netilmicin	70	71.9
Ciprofloxacin	18	38.8
Ofloxacin	30	36.5
Colistin	74	83.5
Cefotaxime	14	
Ceftriaxone	6	
Doxycycline	24	

Similarly, no statistically significant association between biofilm production and metallo beta-lactamase production could be established.

## Discussion

*Pseudomonas aeruginosa* and *Acinetobacter* spp. are common causes of life threatening infections mainly in hospitalized patients. Further, the biofilm production and the metallo-β-lactamase production among these bacteria have further aggravated the problem. Infections with multidrug resistant *P. aeruginosa* and *Acinetobacter* spp. are of serious concern mainly in admitted patients [7].

In this study, 16.4% of *P. aeruginosa* were metallo β-lactamase producers which was in agreement with the finding by Kali et al. (16.3%) [10]. Similar rate of MBL production among *Acinetobacter* spp. as in our study was also reported by Lee et al. (15.1%) [11].

Polymyxin B and colistin have been reported to demonstrate reasonable success in treatment of infections caused by MBL producing *Acinetobacter* spp. and *P. aeruginosa.* However, due to their high toxicity, polymyxins are used for the treatment of only serious infections caused by pan-resistant Gram negative bacilli [12]. A study in Thailand showed that all the multidrug resistant *P. aeruginosa* and *Acinetobacter* spp. were susceptible to polymyxin B and colistin [13].

Very high rates of biofilm production in comparison to our study were reported by Rewatkar and Wadher among the strains of *P. aeruginosa* (90% by Congo red agar method and 83.33% by tube adherence method) [14] and by Badave and Kulkarni among the isolates of *Acinetobacter* spp. (65.2%) [15].

In our study, no statistically significant association between biofilm production and metallo beta-lactamase production along with antimicrobial resistance could be established, which was in contrast to the findings by Heydari and Eftekhar [16] and Singhai et al. [17]. However, higher rates of drug resistance were seen among biofilm producers in comparison to biofilm non producers. Though higher numbers of biofilm producers were detected by tube adherence method in comparison to Congo red agar method but the correlation was statistically insignificant. The lack of significance might be due to small sample size taken in our study.

Though the higher numbers of biofilm producers were detected by tube adherence method in our study, due to the simplicity and cost effectiveness of the Congo red agar method [8] in comparison to tube adherence method, it is more appropriate for laboratory use in the developing countries like Nepal. In addition, its sensitivity and specificity have been reported to be 89% and 100% respectively, when compared to polymerase chain reaction as standard [9]. Similarly, those for tube adherence method were 100% [9].

## Limitations of the study

Due to lack of resources we could not use molecular techniques in our study. Further, this is a uni-center study; multi-center study with large sample size would have generated more reliable results.

## Conclusions

In our study, biofilm production and metallo beta-lactamase production were observed among *Pseudomonas aeruginosa* and *Acinetobacter* spp. However, no statistically significant association could be established between biofilm production and metallo beta-lactamase production.

## Abbreviations

MBL: metallo beta-lactamase production
EDTA: ethylenediaminetetraacetic acid
